# Structure and Genetic Content of the Megaplasmids of Neurotoxigenic *Clostridium butyricum* Type E Strains from Italy

**DOI:** 10.1371/journal.pone.0071324

**Published:** 2013-08-15

**Authors:** Angelo Iacobino, Concetta Scalfaro, Giovanna Franciosa

**Affiliations:** Department of Food Safety and Veterinary Public Health, Istituto Superiore di Sanità, Rome, Italy; Aarhus University, Denmark

## Abstract

We determined the genetic maps of the megaplasmids of six neutoroxigenic *Clostridium butyricum* type E strains from Italy using molecular and bioinformatics techniques. The megaplasmids are circular, not linear as we had previously proposed. The differently-sized megaplasmids share a genetic region that includes structural, metabolic and regulatory genes. In addition, we found that a 168 kb genetic region is present only in the larger megaplasmids of two tested strains, whereas it is absent from the smaller megaplasmids of the four remaining strains. The genetic region unique to the larger megaplasmids contains, among other features, a locus for clustered regularly interspaced short palindromic repeats (CRISPR) and CRISPR associated (cas) genes, i.e. a bacterial adaptive immune system providing sequence-specific protection from invading genetic elements. Some CRISPR spacer sequences of the neurotoxigenic *C. butyricum* type E strains showed homology to prophage, phage and plasmid sequences from closely related clostridia species or from distant species, all sharing the intestinal habitat, suggesting that the CRISPR locus might be involved in the microorganism adaptation to the human or animal intestinal environment. Besides, we report here that each of four distinct CRISPR spacers partially matched DNA sequences of different prophages and phages, at identical nucleotide locations. This suggests that, at least in neurotoxigenic *C. butyricum* type E, the CRISPR locus is potentially able to recognize the same conserved DNA sequence of different invading genetic elements, besides targeting sequences unique to previously encountered invading DNA, as currently predicted for a CRISPR locus. Thus, the results of this study introduce the possibility that CRISPR loci can provide resistance to a wider range of invading DNA elements than previously appreciated. Whether it is more advantageous for the peculiar neurotoxigenic *C. butyricum* type E strains to maintain or to lose the CRISPR-cas system remains an open question.

## Introduction

The *Clostridium butyricum* species is one of the six phylogenetic clostridia Groups whose members may possess the ability to produce the botulinum neurotoxin (BoNT), i.e. the powerful protein toxin causing the neuroparalytic disease of botulism in man and animals. In fact, while most *C. butyricum* strains are non-neurotoxigenic, rare botulinum neurotoxigenic *C. butyricum* strains have been isolated that produce BoNT type E (BoNT/E), one of the seven (A to G) known BoNT serotypes [1].

Conventionally, the BoNT production ability has been ascribed to the *C. botulinum* species, which consists of highly heterogenic clostridia. The heterogeneity of *C. botulinum*, along with the first isolations of botulinum neurotoxigenic *C. butyricum* type E and *C. barati* type F strains [2], [3], and the recognition of a new BoNT/G-producing species (namely, *C. argentinense*) [4], led to the present classification of the BoNT-producing clostridia in six phylogenetic Groups. Each Group of highly related clostridia also includes strains that are non-neurotoxigenic. According to this classification, botulinum neurotoxigenic *C. butyricum* type E and non-neurotoxigenic *C. butyricum* strains constitute clostridia Group VI [5].

We recently showed that each of ten neurotoxigenic *C. butyricum* type E strains, of which six were from Italy and the remaining four were from China, harbors a differently-sized megaplasmid whose structure was proposed to be linear [6]. A ß-lactamase gene was detected in the megaplasmid of eight strains associated to human botulism, whereas it was absent from the megaplasmid of the two remaining strains of environmental origin, suggesting that the ß-lactamase encoding megaplasmids may confer a benefit to their microbial hosts in a clinical environment. However, the ubiquitous presence of the megaplasmids in all analyzed neurotoxigenic *C. butyricum* type E strains, including the two environmental strains that lack the ß-lactamase gene, would suggest that the megaplasmids have additional functions in their microbial hosts, besides conferring antibiotic resistance.

In this study, we focused on the megaplasmids of the six neurotoxigenic *C. butyricum* type E strains isolated thus far in Italy (Table 1) [2], [6]–[9]. Following macrorestriction with *Xho*I and *Sma*I enzymes and pulsed-field gel electrophoresis (PFGE) analysis, the six strains (ISS-20, ISS-21, ISS-86, ISS-109, ISS-145/1, ISS-190) had previously been grouped in two distinct but closely related PFGE clusters, consistent with the two sizes of the megaplasmid in the strains: in the first cluster (strains ISS-20, ISS-21, ISS-109, ISS-145/1) the megaplasmid was about 610 kb, and in the second cluster (strains ISS-86 and ISS-190), the megaplasmid was about 825 kb [6]. The fact that the two PFGE clusters of strains shared a high (>90%) level of similarity [6] suggested a clonal origin of the *C. butyricum* type E strains and led us to suppose that most of the PFGE pattern diversity between the strains might arise from their differently-sized megaplasmids. Therefore, we sought to more thoroughly analyze and compare the megaplasmids of the six *C. butyricum* type E strains, to identify any conserved and/or recently acquired genetic regions that may play crucial roles in the biology of these peculiar microorganisms.

**Table 1:** Botulinum neurotoxigenic *C. butyricum* type E strains used in this study.

Strain^1^	Type of botulism	Year of isolation	Megaplasmid size^2^	PFGE group^2^
ISS-20	Infant botulism	1984	>610 kb	1
ISS-21	Infant botulism	1985	>610 kb	1
ISS-86	Adult intestinal toxemia	1995	∼ 825 kb	2
ISS-109	Adult intestinal toxemia	1996	>610 kb	1
ISS-145/1	Foodborne botulism	1999	>610 kb	1
ISS-190	Infant botulism	2001	∼ 825 kb	2

1 References 2, 6, 7, 8, 9.

2 Reference 6.

## Results and Discussion

### Genetic Maps of the Differently-sized Megaplasmids

**Figure 1:**
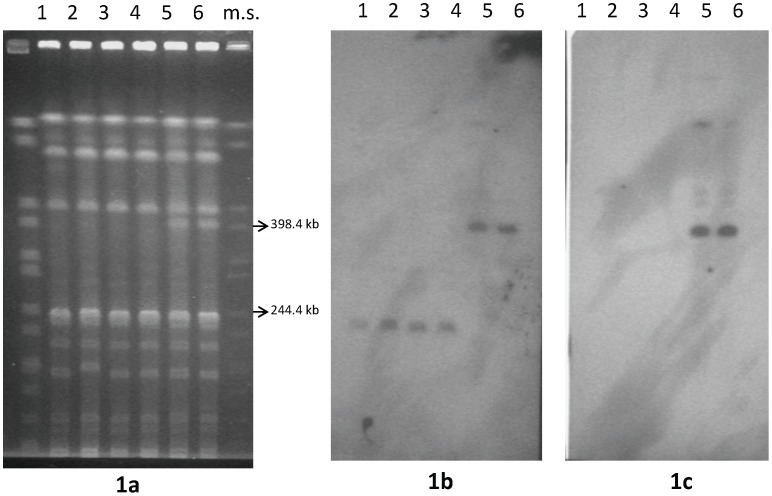
PFGE patterns of *C. butyricum* type E strains digested with *Nru*I restriction enzyme (1a) and Southern hybridization analysis using gene probes *dp* (1b) and *S1* (1c). Strains: ISS-20 (lane 1); ISS-21 (lane 2); ISS-109 (lane 3); ISS-145/1 (lane 4); ISS-86 (lane 5); ISS-190 (lane 6). m.s. (lane 7): molecular standard, *Xba*I-digested DNA from *Salmonella* Braenderup strain H9812 [13].

Most attempts to isolate the megaplasmid bands from Seakem or low-melting point PFGE agarose gels for subsequent restriction enzyme mapping were unsuccessful, possibly because of the low DNA yields and/or because of the unusual conformation of the large DNA molecules determined by the PFGE run conditions [10]. Therefore, we subjected the total genomic DNA of the *C. butyricum* type E strains to restriction analysis and PFGE to select the enzyme(s) yielding the smallest number of clear differences between the PFGE profiles of the two clonal groups of strains. Unexpectedly, *Bam*HI did not digest genomic DNA from any of the *C. butyricum* type E strains, possibly indicating some methylation at the *Bam*HI restriction sites (5′ GGATCC 3′). Cytosine methylation at the internal GATC sites was confirmed by the fact that the DNAs were resistant to digestion with *Sau*3AI, which is unable to cut GATC sequences with methylated cytosine, whereas they were digested by *Mbo*I, which cuts the GATC sites regardless of cytosine methylation (data not shown) [11]. Among the other restriction enzymes tested, *NruI* produced the most straightforward results. The *Nru*I profiles of the six *C. butyricum* type E strains were very similar, except for the presence of a ∼ 400 kb band in the *Nru*I profiles of the two strains (ISS-86 and ISS-190) possessing the larger (∼ 825 kb) megaplasmid. The band was absent from the *Nru*I profiles of the remaining four strains (ISS-20, ISS-21, ISS-109, ISS-145/1) possessing the smaller (∼ 610 kb) megaplasmid (Figure 1a).

To investigate whether the ∼ 400 kb *Nru*I band in the profiles of strains ISS-86 and ISS-190 resulted from the restriction of their megaplasmids, we examined contig 1 (http://www.ncbi.nlm.nih.gov/nuccore/NZ_ACOM01000001.1) of the draft genome sequence available for a *C. butyricum* type E strain isolated in Italy [12]. Contig 1 has previously been shown to partially match the megaplasmid sequences, based on the fact that a β-lactamase gene probe designed from the contig hybridized to the megaplasmid bands of all six *C. butyricum* type E strains [6]. Unfortunately, a full correspondence between contig 1 and the megaplasmid of a specific *C. butyricum* type E strain from Italy could not be established: i) because of the discrepancy between the molecular size of the contig 1 sequence (∼ 758 kb) and the approximate sizes determined by PFGE for the megaplasmids (∼ 610 kb and ∼ 825 kb) [6]; and ii) because the Italian *C. butyricum* type E strain from which the contig 1 sequence has been obtained is labeled as BL5262, which does not correspond to the strain cataloguing system used in our Institute, where the strain had originally been isolated [2], [6]–[9]. Despite the limitation, contig 1 provided a reference sequence for predictive restriction mapping and hybridization studies, as detailed below.

**Figure 2:**
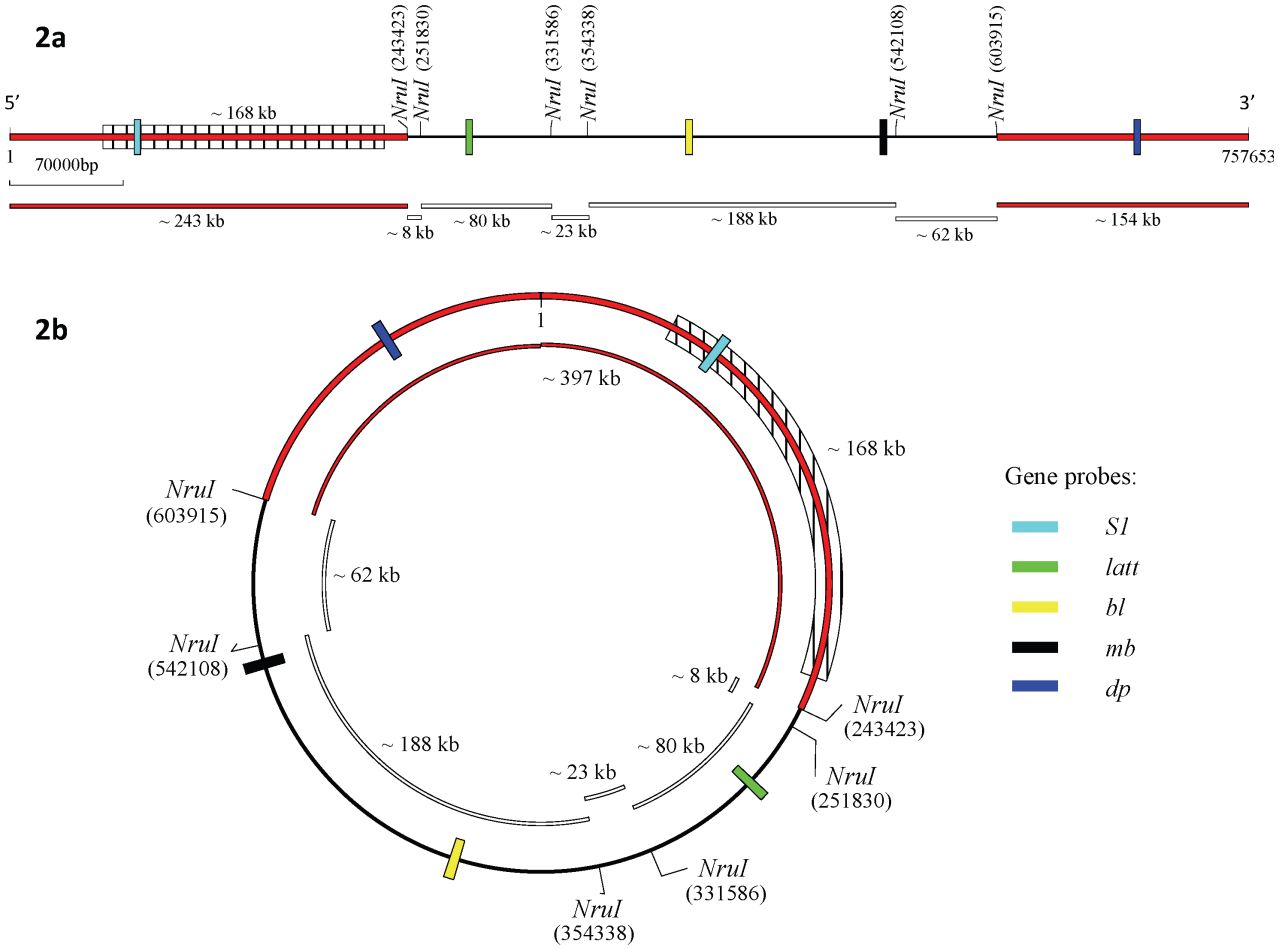
*Nru*I restriction map analysis of the *C. butyricum* type E strain BL5262 contig 1 sequence (http://www.ncbi.nlm.nih.gov/nuccore/NZ_ACOM01000001.1) (757,653 bp) as a linear DNA molecule (2a) and as a circular DNA molecule (2b), and relative positions of the *S1*, *latt*, *bl*, *mb*, and *dp* gene probes. The shaded area (∼ 168 kb) corresponds to the genetic region missing from the smaller megaplasmids of strains ISS-20, ISS-21, ISS-109, ISS-145/1.

The predictive restriction mapping of contig 1 performed by the program DNAMAN (http://www.lynnon.com/) identified six *Nru*I cut sites, resulting in seven restriction fragments of approximately 243 kb, 8 kb, 80 kb, 23 kb, 188 kb, 62 kb, and 154 kb**,** if the DNA molecule analysis was linear (Figure 2a); or six restriction fragments of approximately 8 kb, 80 kb, 23 kb, 188 kb, 62 kb, and 397 kb, if the DNA molecule was circular (Figure 2b). The predictive restriction analysis indicated that a ∼ 400 kb *Nru*I restriction fragment could only result if the sequence was a circular DNA molecule, assuming that the contig 1 sequence corresponded to one of the megaplasmids.

DNA probes targeting four different β-lactamase genes that have dispersed locations in contig 1 were designed and used in Southern hybridization experiments (Table 2 and Figure 2). All gene probes hybridized to the megaplasmid bands of the unrestricted DNAs from the six *C. butyricum* type E strains, confirming that the related β-lactamase genes were carried within the megaplasmids of strains, and also that the megaplasmids do share some conservation, regardless of their different sizes (data not shown). The same size *Nru*I restriction fragments hybridized to the gene probes *latt*, *bl* and *mb* (Table 2) in all six *C. butyricum* type E strains. Specifically, an 80 kb *Nru*I restriction fragment hybridized to the *latt* probe, whereas a 188 kb *Nru*I restriction fragment hybridized to both *bl* and *mb* probes (data not shown). The sizes of the hybridizing *Nru*I fragments were consistent with those of the *Nru*I fragments expected to contain the three distinct β-lactamase genes, as predicted by the restriction map from the contig 1 sequence (Figure 2). These results indicated that the two differently-sized megaplasmids of the six strains share the contig 1 sequence encompassing the *latt*, *bl* and *mb* genes.

**Table 2:** Gene specific probes and primer sets within the *C. butyricum* type E strain BL5262 contig 1 sequence (http://www.ncbi.nlm.nih.gov/nuccore/NZ_ACOM01000001.1) (757,653 bp).

Gene (*probe*)	Gene starting and endingpositions on contig 1	Primer names	Sequence (5′ → 3′)	Amplicon size (bp)
DNA-binding response regulator (*S1*)	76,523–77,206	S1	ACTGCCCATATGGCAAGCTGT	297
		S2	AGGTATTCATACAGTTGCTGG	
Beta-lactamase (*latt*)	278,991–280,007	Latt1	ATGGGGAGAACGTCATAC	408
		Latt2	TTGCCGTCATAGTGAGGT	
Beta-lactamase (*bl*)	413,463–415,439	BL1	ATTCTGTACCAGGGGCATCAG	533
		BL2	TCGCACTAGGCTATTGCTCAT	
Metallo-beta-lactamase (*mb*)	532,357–533,163	MB1	ATTGCGGTGAACCAGCAGTAT	275
		MB2	TGGAGAACAGATCTTAACTCC	
Beta-lactamase domain protein (*dp*)	687,647–688,813	DP1	AGCGGCATCTGCTGTATTTCC	360
		DP2	ATGGCGCCAATGGTTCACTGG	

According to the predicted restriction map of contig 1, the remaining β-lactamase domain protein (*dp*) gene probe, which is located close to the 3′-end of the contig 1 sequence, should lie within a 154 kb *Nru*I restriction fragment if the DNA molecule was linear (Figure 2a), or within a 400 kb *Nru*I restriction fragment if the DNA molecule is circular (Figure 2b). Consistent with the latter hypothesis, the *dp* gene probe hybridized in Southern blots to the ∼ 400 kb *Nru*I restriction fragment that was only visible in the profiles of strains ISS-86 and ISS-190, both carrying the larger megaplasmids (Figure 1b). These results indicated that the contig 1 sequence corresponded to the larger megaplasmid sequences of the *C. butyricum* type E strains ISS-86 and ISS-190 (Table 1), and provided a further indication that those megaplasmids have a circular structure.

The *dp* gene probe hybridized to a ∼ 250 kb *Nru*I restriction fragment in the four remaining *C. butyricum* type E strains (ISS-20, ISS-21, ISS-109, and ISS-145/1), all carrying the smaller megaplasmid (Figure 1b). Considering that fragment length estimates by PFGE are not precise (in our case, estimates were obtained by comparison to *Xba*I-digested DNA from *Salmonella* Braenderup strain H9812) [13], the ∼ 150 kb size difference observed between the *dp* positive *Nru*I restriction bands (∼400 kb and ∼ 250 kb, respectively) from the two clusters of *C. butyricum* type E strains was similar to the estimated ∼ 215 kb size difference between the larger (∼ 825 kb) and the smaller (∼ 610 kb) megaplasmids [6]. These results suggested that most of the ∼150 kb genetic region missing from the genomic DNAs of *C. butyricum* type E strains ISS-20, ISS-21, ISS-109, and ISS-145/1 was from the megaplasmid.

To verify the above findings, we designed another gene probe (*S1*) from the 5′-end of the contig 1 sequence, as opposite to the *dp* gene probe located close to the 3′- end (Table 2, Figure 2a). According to the predicted restriction map of the contig 1 sequence, the *S1* gene probe should lie within a 243 kb *Nru*I restriction fragment if the DNA molecule is linear, or within the same 400 kb *Nru*I restriction fragment hybridizing to the *dp* probe if the DNA molecule is circular (Figure 2b). The Southern experiments revealed that in strains ISS-86 and ISS-190 the *S1* gene probe hybridized to the same 400 kb *Nru*I fragment that also hybridized to the *dp* gene probe (Figure 1c). Southern hybridization of *dp* and *S1* gene probes to genomic DNAs of strains ISS-86 and ISS-190 restricted with other enzymes also produced identical hybridization patterns, i.e. both the *dp* and *S1* gene probes hybridized to the same restriction fragments, whose sizes were consistent with those predicted by the restriction analysis of contig 1 (data not shown). These results indicated that the contig 1 sequence of strain BL5262 fully covers the larger megaplasmids sequences. Consequently, the *C. butyricum* type E strain BL5262 should likely correspond to either *C. butyricum* type E strains ISS-86 or ISS-190 (Table 1). The results confirmed that the two *dp* and *S1* gene probes are closely located in a circular DNA molecule (Figure 2b), rather than residing on the opposite ends of a linear DNA molecule (Figure 2a), and consequently that the larger megaplasmids are circular rather than linear, as previously proposed [6]. Circularity was also indicated by the fact that the 5′- and 3′-terminal ends of the contig 1 sequence (http://www.ncbi.nlm.nih.gov/nuccore/NZ_ACOM01000001.1) perfectly matched, which is expected for a closed contiguous circular sequence.

The *S1* gene probe did not hybridize to any *Nru*I or other enzymes restriction fragments from the four *C. butyricum* type E strains (ISS-20, ISS-21, ISS-109, ISS-145/1) carrying the smaller (∼ 610 kb) megaplasmids (Figure 1c). These results showed that the latter strains lack the genetic region at the 5′-end of the contig 1 sequence where the *S1* gene probe was designed (Figure 2a). The ∼250 kb *Nru*I fragment from these strains that hybridize to the *dp* gene probe would therefore correspond to the 400 kb *Nru*I fragment of strains ISS-86 and ISS-190, but missing a ∼150 kb segment. This inference implies that the smaller megaplasmids of strains ISS-20, ISS-21, ISS-109, and ISS-145/1 also possess a circular structure.

The current evidence of circularity of the megaplasmids supplants our previous interpretation of linearity, which was based on accurate but incomplete information from PFGE analyses [6].

### Analysis of the Genetic Region Common to the Megaplasmids

As shown above, the differently-sized megaplasmids share a genetic region containing four different ß-lactamase genes. Genes conferring resistance to quaternary ammonium compounds, oxenatocin A, acriflavin, and toxic ions are also present within the common region of megaplasmids, as inferred from analysis of the contig 1 sequence of strain BL5262. Besides, the region includes a number of putative cell surface structural genes, as well as several metabolic genes; microbial cell surface components might play a role in the bacteria interaction with the environment, whereas metabolism genes expand the clostridia metabolic abilities and likely allow them to rapidly adapt to changing environments. Notably, the shared genetic region is also particularly rich in transcriptional regulators of different families and two-component regulatory systems. Finally, it contains numerous insertion sequence elements/transposons, suggesting that multiple transposition events leading to genetic rearrangements might have contributed to the megaplasmids formation during evolution.

### Characterization of the Extra Genetic Region of the Larger Megaplasmids

To more precisely define the genetic region of the larger megaplasmids that was missing from the genomes of the strains carrying the smaller megaplasmids, we performed a BLAST comparison (http://blast.ncbi.nlm.nih.gov/Blast.cgi) between the contig 1 sequence from the *C. butyricum* type E strain BL5262, corresponding to the larger megaplasmid of either ISS-86 or ISS-190 strains, and the whole genome draft sequence of another *C. butyricum* type E strain from Italy, labeled as 5521 (http://www.ncbi.nlm.nih.gov/nuccore/NZ_ABDT00000000.1) [12]. Strains BL5262 and 5521 differed in genome size by about 218 kb (4.758 kb and 4.540 kb, respectively), so we assumed that they represented two different PFGE clusters.

BLAST comparison revealed that a 168,328 bp genetic region present in the contig 1 (i.e., the megaplasmid) of strain BL5262 was totally missing from the whole genome draft sequence of strain 5521. The region spanned position 61,674 to 230,001 bp of the contig 1 sequence, thus accounting for 77% of the ∼ 218 kb difference in genome size of the two strains. The whole ∼ 168 kb genetic region was flanked at both 5′- and 3′- ends by an integrase core domain protein and a transposase, indicating that it is a potentially mobile genetic element. The region harbors 151 coding sequences, including enzymatic and metabolism proteins, structural proteins, chemotaxis related proteins and regulatory proteins; it also contains a cassette of clustered regularly interspaced short palindromic repeats (CRISPR) and CRISPR associated (*cas*) genes. No other CRISPR loci were identified in the genome draft sequences of strains BL5262 and 5521.

The CRISPR-cas system is a recently discovered adaptive immune system of many bacteria. It consists of highly conserved short repeat sequences separated by hypervariable short spacer sequences, the latter acquired from invading genetic elements and inserted next to a leader sequence. The CRISPR cassette is transcribed into RNA molecules that, in combination with the Cas proteins, recognize, bind and inactivate the invading DNA, thus preventing subsequent infection [14], [15]. The *cas*1 gene, encoding a DNAse involved in acquiring the spacer sequences in a CRISPR cassette, is universally present in bacteria possessing a functional CRISPR-*cas* locus [15]. We designed specific primers (*cas1_forward*: 5′ AGCAATCTAGTTTTATTGCAG 3′; and *cas1_reverse*: 5′ AGTGGAACTTTCTATCCAAGA 3′; located between 178,981 and 179,698 bp of the contig 1 sequence) targeting the *cas*1 gene of the CRISPR-*cas* locus of the *C. butyricum* type E strain BL5262, in order to PCR screen our six *C. butyricum* type E strains for the presence of the gene. PCR products of the expected size (718 bp) were obtained from strains ISS-86 and ISS-190, harboring the larger megaplasmid; in contrast, no *cas*1 gene PCR product was obtained from strains ISS-20, ISS-21, ISS-109, and ISS-145/1, which harbor the smaller megaplasmid and lack the 168,328 bp genetic region (data not shown). These results confirmed the presence of the CRISPR-*cas* locus in the larger, but not the smaller megaplasmid.

### Analysis of the CRISPR-cas Loci

**Figure 3:**
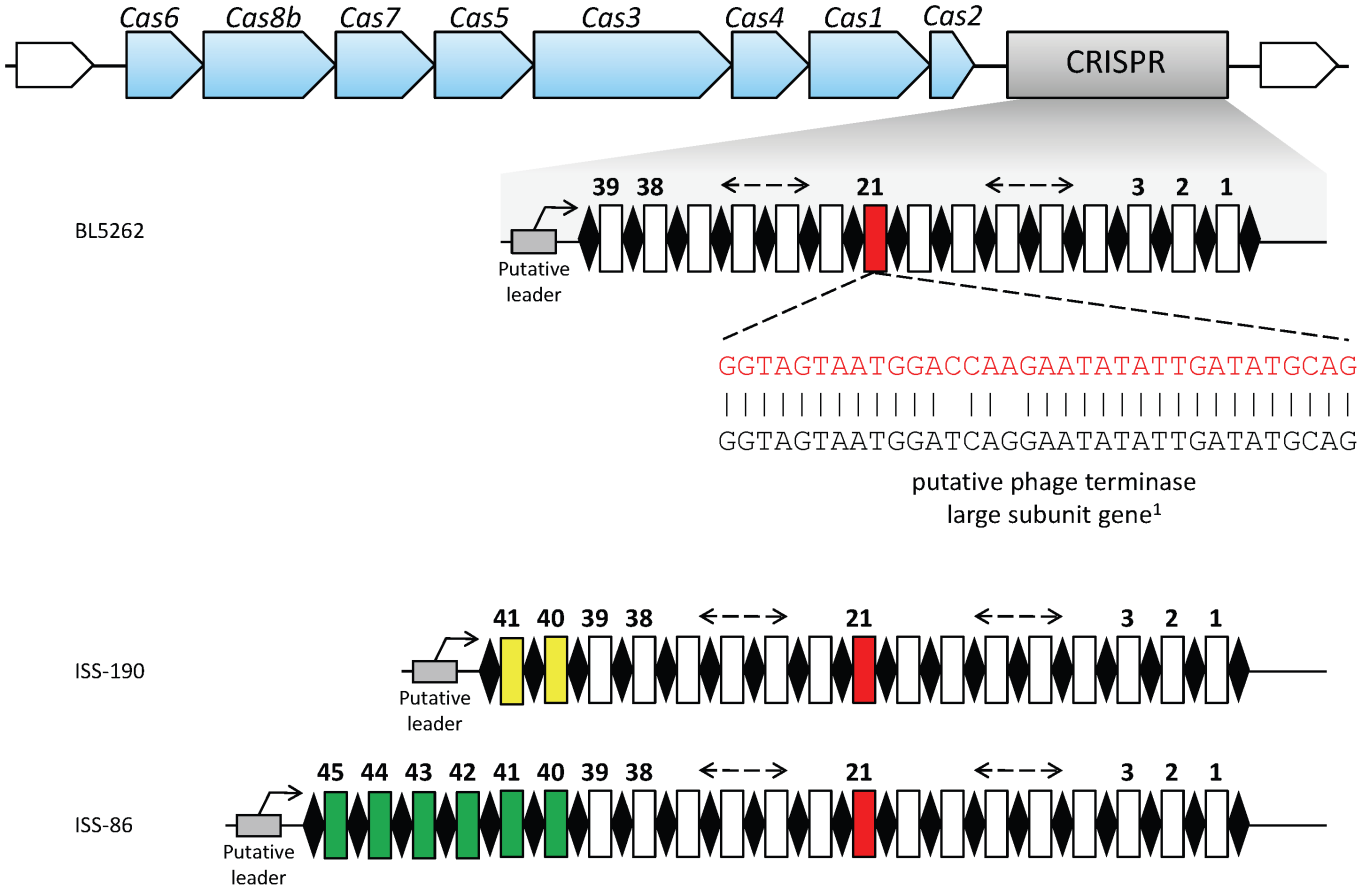
Organization of the CRISPR-*cas* locus in contig 1 (http://www.ncbi.nlm.nih.gov/nuccore/NZ_ACOM01000001.1) of *C. butyricum* type E strain BL5262. The whole CRISPR-*cas* locus is 11,514 bp (nucleotides 175,848 through 187,362 of contig 1). ♦ CRISPR conserved repeat sequences. CRISPR hypervariable spacer sequences numbered according to their acquisition order, with the more recently added spacers having the highest numbers. The additional distinct spacers of strains ISS-190 and ISS-86 are coloured in yellow and green, respectively. ^1^The 21st CRISPR spacer sequence matches a conserved sequence from putative phage terminase large subunit genes within the genomes of *C. botulinum* type E strain Alaska E43 and *C. botulinum* type B strain Eklund 17B (see Table 3).

We then analyzed the organization of the CRISPR-*cas* locus in contig 1 of *C. butyricum* type E strain BL5262 (Figure 3). According to the most recent classification, the *cas* gene order and the *cas* signature genes (namely, *cas*3 and *csh*1 or *cas8b*) are consistent with CRISPR-*cas* subtype I-B [16]. Downstream of the *cas*2 gene, the presence of a CRISPR cassette at positions 175,848 through 178,419 of contig 1 was revealed by the in silico analysis of the contig performed by the program CRISPRFinder (http://crispr.u-psud.fr/Server/) [17].

Based on the flanking regions of the CRISPR cassette in contig 1 of strain BL5262, a primer pair was designed to PCR amplify and sequence the CRISPR cassettes of strains ISS-86 and ISS-190 (GenBank accession numbers of the obtained sequences: KF150773 and KF150772, respectively). Comparative analysis of the CRISPR sequences from the three strains revealed that they shared the same repeats of 30 bp, separated by 39 identical spacers of about 35 bp (33–37 bp) in the same order. However, compared to the CRISPR sequence of strain BL5262, the CRISPR sequences of strains ISS-86 and ISS-190 contained six and two additional distinct spacers, respectively, which were located at the same end of the CRISPR array, where the leader sequence was assumed to be present (Figure 3).

The high number of spacer sequences and the presence of the *cas*1 gene, required for spacer acquisition [15], indicate that the CRISPR-cas system of the microorganisms is likely to be functional. Moreover, the presence in the three CRISPR cassettes of 39 shared consecutive leader-distal (old) spacers supports that the *C. butyricum* type E strains BL5262, ISS-86 and ISS-190 coevolved from a common ancestor. On the other side, the occurrence of several diverse leader-proximal (new) spacers in the CRISPR arrays of strains ISS-86 and ISS-190 likely depends on these microorganisms most recent contacts with different foreign DNA elements. In the attempt to establish a correspondence between strain BL5262 and one of the Italian strains of this study, we hypothesize that BL5262 and ISS-190, sharing the most similar CRISPR spacer contents, could be two individual isolates recovered from the same infant botulism patient [8] (Table 1): in fact, in a recent study on the human gut microbiome, Stern *et al.* showed that the CRISPR arrays of gut bacteria evolve fast by acquiring new spacers within the human intestine, as a result of phage - bacteria constant interactions [18]. The higher number of recently acquired new spacers in strain ISS-86 can be attributed to a more prolonged activity of its CRISPR locus: this strain was isolated from a distinct case of intestinal toxemia botulism in an adult [7] (Table 1). Altogether, these findings suggest that BL5262, ISS-86 and ISS-190 are independent isolates of the same bacterial strain, rather than different strains.

To identify potential phage- or plasmid-targeting sequences, we compared each CRISPR spacer sequence from the three *C. butyricum* type E isolates to existing sequences in GenBank database by BLAST (http://blast.ncbi.nlm.nih.gov/Blast) and by CRISPRTarget (http://brownlabtools.otago.ac.nz/CRISPR_WEB/crispr_analysis.pl), another recently released software specifically devoted to the analysis of CRISPR targets [19]. Of the 47 CRISPR spacers identified among the three *C. butyricum* type E isolates, 23 showed sequence similarity (≥26 identical residues) to known putative mobile genetic elements, such as phages, prophages, plasmids (Table 3).

**Table 3:** Analysis of the CRISPR spacers in *C. butyricum* type E strains BL5262 (http://www.ncbi.nlm.nih.gov/nuccore/NZ_ACOM01000001.1), ISS-86 (GenBank: KF150773) and ISS-190 (GenBank: KF150772).

Spacer number^1^	Spacer sequence^3^	Nucleotide identity	Target description	Genome accession number
1	AT CTTTCTAATCATAATTGTTTCAATGTCTTCTAAT	28/36	*Staphylococcus* phage phi2958PVL	AP009363
2	ATAA AACTAGAAAAAAAGATGATGTTGAATTTGAT	27/35	*Clostridium* phage phiCD27	EU719189
			*Clostridium* phage phiC2	DQ466086
4	CTACATGTATTTTTTACACCTTCCCATGTTTCACC	26/35	*Bacillus* phage BCU4	JN797798
5	GTTGGAGAATCTGTTGTGCTAAATTTATTACTACT	26/35	*Campylobacter* phage vB_CcoM-IBB_35	HM246721
			*Campylobacter* phage CPt10	FN667789
			*Campylobacter* phage CP220	FN667788
6	TTA GAAAAAGATTTAGATAGTAGGATATGGGAAGA	26/35	*Acinetobacter* phage ZZ1	HQ698922
7	GCAAATTTATTCTCTAATGCATTAAAAGCACTTGGC	27/36	*Clostridium botulinum* B str. Eklund 17B(hypothetical phage protein)	NC010674
13	AGAAGTGGACTAGAAGAAGTAGTAAATAAATTAA	26/34	*Bacillus* phage Bastille	JF966203
14	GATGATGATAATAATGATGATGATAAGCTATTTAA	28/35	*Lactococcus* phage 949	HM029250
15	TTTT CATTTTCACATTTTAAGCCATACAGTAAACC	28/35	*Borrelia burgdorferi* 29805 plasmid	NC012498
17	AT GCATAAATATTATCTCTATATTTAATTATAAC	26/34	*Campylobacter* phage vB_CcoM-IBB_35	HM246723
18	GCAT TAGCACTATTAGCATTAGAATTACAATCTA	26/34	*Lactococcus* phage bIL309	AF323670
19	ATCTGAACAGCATCATTAATTGCTAAATCTGTAAGG	26/36	*Colwellia* phage 9A	HQ317390
21	GGTAGTAATGGA CCAAGAATATATTGATATGCAG	32/34	*Clostridium botulinum* E3 str. Alaska E43(prophage)^4^	NC010723
			*Clostridium botulinum* B str. Eklund 17B(prophage)^4^	NC010674
27	GC AGTAATATTTGCTCATTTTTCAAATAATATTAAAC	29/37	*Clostridium botulinum* E3 str. AlaskaE43(prophage)^4^	NC010723
30	TTGCACTT TTTGTAATAATCCATGTATTACCACTCTT	29/37	*Bacillus cereus* B4264 (hypothetical phage protein)	NC011725
31	ATTTAAATTTAACTTAAAATATAAATTTGGTCCTAA	26/36	*Campylobacter* phage vB_CcoM-IBB_35	HM246720
34	GAATACTAAAAATGTAAGTGAATTGCAGATTTTATA	26/36	Enterobacteria phage vB_KleM-RaK2 (*Klebsiella*)	JQ513383
35	AT GGAAAATAAATTAAATGAACTTAATTCTGTAT	26/34	*Clostridium* phage c-st	AP008983
37	AA CGATAAATTTTCAACATAAGTATTTCTTAATC	29/34	*Clostridium* phage phiSM101	CP000315
	AA CGATAAATTTTCAACATAAGTATTTCTTAATC	29/34	*Clostridium cellulovorans* 743B (prophage)^4^	NC014393
38	ACTGAAAAATTGAAATCAACTTGGATTTCCAAATCT	26/36	Enterobacteria phage vB_KleM-RaK2 (*Klebsiella*)	JQ513383
40^2^	TTGCTTTT CCCTCTTCAATAAGTTCTTTCTGACTC	28/35	*Acinetobacter* phage Ac42	HM032710
43^2^	ATATTTGA TATTCTAAACAATTGTGTACCTGATGG	26/35	*Lactococcus* phage P087	FJ429185
44^2^	TTATTTATATAAACCATTTTTATCACCTCTAAGTTA	26/36	*Pseudomonas* phage PA7	JX233784
			*Pseudomonas* phage phiKZ	AF399011
	TT ATTTATATAAACCATTTTTATCACCTCTAAGTTA	26/36	*Campylobacter* phage CP81	FR823450

1 Spacers are numbered according to their acquisition order, i.e. the more recently added spacers have the highest numbers.

2 Spacers 40, 43 and 44 are those of the CRISPR array of strain ISS-86.

3 Bold underlined nucleotides match the target sequences.

4 Putative prophage sequences within bacterial genomes were identified through the program Prophinder (http://aclame.ulb.ac.be/prophinder).

**Figure 4:**
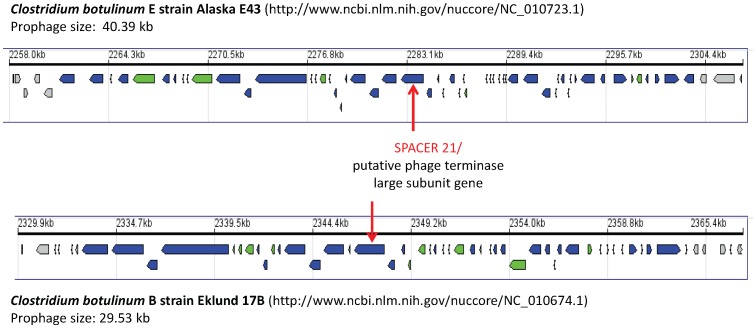
Putative prophages containing the phage terminase large subunit gene matching the 21st CRISPR spacer that were identified within the genomes of *C. botulinum* strains Alaska E43 and Eklund 17B by the prophage prediction program Prophinder (http://aclame.ulb.ac.be/prophinder).

Intriguingly, the best match was exhibited by the twenty-first 34-bp CRISPR spacer, present in all three isolates, which targeted a 32 bp fragment of a putative phage terminase large subunit gene within the genomes of two different *C. botulinum* strains, i.e. *C. botulinum* type E strain Alaska E43 and non-proteolytic *C. botulinum* type B strain Eklund 17B (Table 3 and Figure 3). The two mismatches were at degenerate positions and did not result in amino acid changes in the encoded phage terminase protein: it has been reported that perfect matches are not necessary to provide immunity, especially if mismatches are located at degenerate positions [15]. Submission of the whole genome sequences of the *C. botulinum* strains Alaska E43 (http://www.ncbi.nlm.nih.gov/nuccore/NC_010723.1) and Eklund 17B (http://www.ncbi.nlm.nih.gov/nuccore/NC_010674.1) to the prophage prediction tool Prophinder (http://aclame.ulb.ac.be/prophinder) [20] showed that the phage terminase large subunit genes containing the CRISPR homologous sequence were part of a putative prophage sequence integrated within each of the two strain chromosomes. Although the phage terminase large subunit genes of the two strains were very similar (96% level of identity), their corresponding putative prophages were different: the putative prophage of strain Alaska E43 consisted of 40,391 bp and included 55 coding sequences, whereas the putative prophage of strain Eklund 17B consisted of 29,531 bp and included 45 coding sequences (Figure 4). *C. botulinum* strains Alaska E43 and Eklund 17B belong to clostridia Group II that is closely related to Group VI *C. butyricum* type E [10]. CRISPR spacers of certain bacterial species matching prophage sequences within genomes of genetically close species have recently been described [21].

The unexpected finding of our study was that the twenty-first CRISPR spacer shared 32 nucleotide residues at identical locations with similar coding DNA sequences (CDSs) of two different prophages; remarkably, the second, fifth and forty-fourth CRISPR spacers (the latter only present in isolate ISS-86) also exhibited sequence similarity to analogous CDS of different bacteriophages (Table 3). These findings indicate that the CRISPR locus of the *C. butyricum* type E isolates has incorporated several DNA sequences that are conserved in different invading genetic elements. These CRISPR spacers might not exclusively function as memory of previously encountered invading DNA, as currently thought, but they would rather specifically target a wide range of potential invading genetic elements containing the conserved sequence, while limiting the CRISPR cassette growth. This newly identified strategy of the CRISPR spacers would provide the bacterial host with a more efficient and versatile immune system than previously known.

Additional 22 CRISPR spacers shared ≥26 nucleotide residues with known sequences of phages, prophages or plasmids of clostridia *spp* other than botulinum, or of species from phylogenetically distant genera, such as Bacillus, Lactococcus, Acinetobacter, Staphylococcus, Klebsiella (Enterobacteria), and Campylobacter (Table 3). These bacteria are all permanent or transient members of the human and animal intestinal flora. Since CRISPR spacers are acquired in response to DNA invasion, we hypothesize that the neurotoxigenic *C. butyricum* type E isolates have been exposed to mobile genetic elements harboring these sequences in a human or animal intestinal environment, as previously mentioned. An analogous hypothesis has been formulated for genetically distant lactic acid bacteria harboring similar CRISPR loci [22].

### Conclusions

Our present demonstration that the differently-sized megaplasmids of the *C. butyricum* type E strains from Italy share a genetic region containing four different ß-lactamase genes supports that they are antibiotic-resistance plasmids, as previously reported [6]. A striking feature of the genetic region common to the megaplasmids is that it includes a number of transcriptional regulators and two-component regulatory systems, the latter recently involved in the regulation of expression of the *bont*/A gene [23]. No *bont*/E gene regulation mechanisms have so far been identified, therefore the possibility that some of the shared megaplasmid-encoded putative regulators influence the *bont*/E expression in neurotoxigenic *C. butyricum* type E strains warrants further research.

Our results also showed that: *i*) the two groups of neurotoxigenic *C. butyricum* type E strains from Italy possessing differently-sized megaplasmids vary for a 168 kb genetic region which is only present within the larger megaplasmid of one group; *ii*) the 168 kb genetic region is candidate to lateral gene transfer, since it is flanked by integrase and transposase genes; *iii*) the 168 kb genetic region contains a CRISPR-cas locus; *iv*) this locus is functional, as demonstrated by the identification of additional different spacers in two isolates. The CRISPR-cas locus likely provides the strains possessing it with a defense against invading genetic elements, especially those present in the intestinal environment.

Therefore, the uptake of foreign DNA is expected to differ between the two groups of *C. butyricum* type E strains, depending on the presence of the CRISPR-cas loci in their megaplasmids. To test this hypothesis, it would be of interest to compare the susceptibility of the two groups of *C. butyricum* type E strains to phage infection and/or conjugative plasmid DNA uptake. Unfortunately, phage infection studies in clostridia are hampered by the lack of appropriate methodologies [24] Another obstacle to phage infection and plasmid conjugation experiments is the presence of DNA restriction-modification (RM) system (i.e., another immune system attacking foreign DNA that is unmethylated). We found that all six Italian *C. butyricum* type E strains possessed a DNA restriction-modification (RM) system, as demonstrated by resistance to *Bam*HI and *Sau*3AI restriction. Analysis of the draft genome sequences of *C. butyricum* type E strains BL5262 and 5521 confirmed that contig 5 (http://www.ncbi.nlm.nih.gov/nuccore/ACOM01000005.1) and contig 53 (http://www.ncbi.nlm.nih.gov/nuccore/ABDT01000053.2) of the two strains, respectively, harbored a putative *Sau*3AI RM gene cassette.

The fact that two of the six neurotoxigenic *C. butyricum* type E strains of this study possess multiple mechanisms against invading DNA, i.e. the CRISPR-cas and the RM systems, is not unusual among bacteria. What remains to be clarified is whether it is more advantageous for neurotoxigenic *C. butyricum* type E to maintain both immune mechanisms or to possess only one of them. Strains ISS-86 and ISS-190 possess the larger megaplasmid with the CRISPR-cas locus and also have the chromosomal RM locus, whereas strains ISS-20, ISS-21, ISS-109 and ISS-145/1 do not have the CRISPR-cas locus on the megaplasmid. The evolutionary tradeoff likely depends on a number of contrasting factors, such as the need of acquiring advantageous foreign DNA versus prevention of costly DNA uptake. The study of the mobility of the 168 kb genetic region containing the CRISPR-cas locus will help decipher the role of the CRISPR-cas system in these peculiar microorganisms.

## Materials and Methods

### Strains and Culture Conditions

The six neurotoxigenic *C. butyricum* type E strains used in this study are listed in Table 1. All had been isolated in Italy from distinct cases of human botulism. Their origin and relevant characteristics are detailed elsewhere [2], [6]–[9]. Freeze-dried seeds were inoculated into 9 ml TPGY broth (5% Trypticase, 0.5% peptone, 0.4% glucose, 2% yeast exctract, 1% L-cysteine hydrocloride monohydrate) and grown for 24 h. Isolated colonies were obtained by plating the TPGY broth cultures on egg yolk agar (EYA) plates (Oxoid, Milan, Italy). All cultures were incubated at 35°C in anaerobiosis (GasPack jars, Oxoid) for either 24 h for broth cultures or 48 h for solid cultures.

### PFGE and Southern Blot Analyses

DNA was isolated from single colonies of each strain as previously described [6]. DNA digestion with different restriction enzymes was performed according to the manufacturer instructions (New England BioLabs, Ipswich, MA). PFGE runs were carried out in a contour-clamped homogeneous electric field system (CHEF Mapper apparatus, BioRad Laboratories, Hercules, CA) through 0.8% Seakem Gold agarose gel (Cambrex, East Rutherford, NJ) in 0.5 X Tris-borate-EDTA buffer, at 6 V/cm and 14°C. The PFGE run conditions for separating the digested DNA samples required pulse times increasing from 4 to 40 s (linear ramping factor) over 20 h. The DNA isolated from *Salmonella* serovar Braenderup strain H9812 and digested with *Xba*I was used as the molecular marker [13]. Gels were stained with ethidium bromide and visualized in a GelDoc 2000 apparatus (Bio-Rad).

Primers for the preparation of specific gene probes by the PCR DIG Probe Synthesis kit (Roche Diagnostics GmbH, Mannheim, Germany) are listed in Table 2. Southern hybridizations of pulsed-field gels with the gene probes were performed as previously described [6].

### CRISPR Amplification and Sequencing

Primers *Crispr-for* (5′ AGGTGTTGACAATCTAGTTCC 3′) and *Crispr-rev* (5′ TACCAGCTGATTTTAGGGCGA 3′) were derived from sequences upstream and downstream of the CRISPR region in contig 1 of strain BL5262. Amplification reactions were carried out by using 1 µl of template prepared from strains ISS-86 and ISS-190 as described elsewhere [6], and the proof-reading Phusion High Fidelity DNA polymerase (New England BioLabs) according to the conditions specified by the manufacturer. PCR amplicons were purified (QIAquick PCR purification kit, Qiagen, Milan, Italy). The nucleotide sequences were determined on both DNA strands by Sanger sequencing at Bio-Fab Research, Rome, Italy. Sequences were deposited in GenBank with accession numbers KF150772 and KF150773.

### Bioinformatics Analyses

In silico analyses were performed with the genome sequences of the following strains: neurotoxigenic *C. butyricum* type E strain BL5262 (http://www.ncbi.nlm.nih.gov/nuccore/NZ_ACOM00000000.1 ); neurotoxigenic *C. butyricum* type E strain 5521 (http://www.ncbi.nlm.nih.gov/nuccore/NZ_ABDT00000000.1 ); *C. botulinum* strain Alaska E43 (http://www.ncbi.nlm.nih.gov/nuccore/NC_010723.1); *C. botulinum* strain Eklund 17B (http://www.ncbi.nlm.nih.gov/nuccore/NC_010674.1).

The predictive restriction maps were obtained by the program DNAMAN (http://www.lynnon.com/). The BLAST program (http://blast.ncbi.nlm.nih.gov/Blast.cgi) was used for comparative sequence analyses. The program CRISPRFinder (http://crispr.u-psud.fr/Server/) [17] was used to detect and analyze the CRISPR sequences in the megaplasmid sequence of the *C. butyricum* type E strain BL5262. The CRISPR spacers were assessed for homology to known sequences using BLASTn together with the program CRISPRTarget (http://brownlabtools.otago.ac.nz/CRISPR_WEB/crispr_analysis.pl) [18]. The prophage prediction tool Prophinder (http://aclame.ulb.ac.be/prophinder) [19] was used to identify putative prophage sequences in the genomes of *C. botulinum* strains Alaska E43 and Eklund 17B.
